# The Influencing Factors of Participation in Online Timebank Nursing for Community Elderly in Beijing, China

**DOI:** 10.3389/fpubh.2021.650018

**Published:** 2021-04-08

**Authors:** Yan Wu, Yanran Ding, Cong Hu, Lei Wang

**Affiliations:** ^1^School of Economics, Beijing Technology and Business University, Beijing, China; ^2^School of Economics and Resource Management, Beijing Normal University, Beijing, China

**Keywords:** timebank, elderly care, questionnaire survey, logit model, China

## Abstract

This study uses the logit model through questionnaire data of Beijing in 2019 to investigate the participation willingness of online timebank elderly care, especially to discover different influencing factors on the participation willingness between the youth group and the elderly group. We find that: First, the health status of elderly people and the number of elder families of young people have significant positive impacts on their willingness to participate in online timebank. Second, the experience of participating in voluntary activities has a significant positive effect and it has a far greater impact in the young group than that in the elderly group. Third, the more the free time, the higher the participation willingness in the young group, but it is the opposite in the elderly group. Fourth, the years of education and party member have significant promoting effects on the participation willingness in both groups. Such heterogeneous influencing factors can help develop online timebank nursing for dealing with the increasingly serious population aging problem in China and also other developing countries.

## Introduction

The process of population aging in China is accelerating, especially in big cities. By the end of 2019, the number of elderly people over 60 years old has reached 254 million, accounting for 18.1% of the Chinese total population. Taking the capital city Beijing as an example, the city's registered elderly population above 60 years old is ~3.49 million, accounting for 25.4% of the city's total population. In addition, with the increase of one-child families and a large number of young adults leaving the country to work in cities, the proportion of empty nest families in China has also increased sharply. The proportion of empty-nest elderly in China has reached 49.17% in urban areas and 38.13% in rural areas, which has led to a serious social issue in China.

It should be admitted that the aging population, migration, and empty nest are the global drivers for change and are not specific for China, but the situation in China is very special. Due to the implementation of the family planning and one-child policy for many years, the proportion of young people in China has decreased rapidly, and the feature “old before getting rich” has emerged in China, that is, people's income has not reached a certain level but the aging problem is becoming more and more serious. For example, China's newborn falls abruptly in 2020.1.76 million fewer babies were born in 2020 than that in 2019, a drop of about 15 percent^1^. In addition, due to the lack of labor rights protection and the diligent characteristic, Chinese young people are often overtime at work, leaving little time to care for the elderly families, especially young people who work and live far away from their hometown.

However, Chinese institutional care is difficult to become the main care model of the elderly due to limited numbers and unaffordable fees for most Chinese. This is also the case in some other developing countries like Russia and Argentina. In this context, the new community-based care “timebank” may become a feasible elderly care model and can play an important role in solving this serious problem of public health in China and also other developing countries. “Timebank” means that the participator deposits the time which they spent on taking care of other community elderly in the timebank. When they encounter difficulties in their elderly age in the future, they can withdraw the deposited time and get corresponding time nursing from others (1). In the concept of timebank, time as a currency can be stored and exchanged. The purpose of the timebank is to use the time of payment in exchange for the care of others, and the bank is a channel for the circulation of time.

With the trend of miniaturization of families in China, most people have no enough energy or time to support their parents or other elderly relatives. In 2010, some members of the Chinese People's Political Consultative Conference proposed the establishment of a timebank that uses the community as a unit to serve the elderly in the community. On July 4, 2015, China issued the “Guiding Opinions on Actively Promoting the “Internet +” Action,” which clarified the full play of the role of the Internet in the optimization and integration of social resource allocation in China. Therefore, the online timebank model may play a positive role in improving the elderly care system in the future. Therefore, this study uses logit model through questionnaire data of the youth group and the elderly group to investigate the participation willingness of online timebank elderly care, especially to discover the influence factors as well as the potential problems, so as to propose countermeasures to promote the wide development of online timebank and deal with the increasingly serious population aging problem in China and also other developing countries.

In the next section, we will analyze the mechanism and operation mode of the Chinese timebank and give a literature review. Section The Survey Process and Data Analysis introduces the survey process and makes a short data analysis. Section Methods describes the logit model, variable selection, and model specification. Section Exploring the Influencing Factors of Participation Willingness empirically assesses the effects of different factors on participation willingness in online timebank. Section Discussions discusses the results based on the interview information. Finally, section Conclusions and Policy Implications provides the conclusion and policy implications.

## Timebank Mechanism and Literature Review

### Timebank Mechanism in China

Timebank is different from the ordinary bank because it is not money, but the time that is deposited and withdrawn. Through this mechanism, people can not only store time but also consume the stored time. After years of development, timebank has gradually been used in community mutual assistance scenarios, mainly retired young elderly to provide daily assistance to the elderly to store time, and then withdraw time when they need healthcare in the future and receive the same time service. In general, the timebank can alleviate the huge pressure on the only child to support their parents in China and provide a new community healthcare method for themselves to meet the needs in the future. Relying on the community, timebank is conducive to creating a more harmonious community-neighbor relationship. By using the internet platform to provide more accurate nursing healthcare services and medical services for the elderly, in addition to integrating the community, online timebank can also integrate with elderly care institutions and medical institutions. Therefore, online timebank is a useful platform that can better match the demand side and supply side of elderly healthcare services.

First, under the traditional concept, for the elderly who need nursing care, it is generally based on family or relatives' nursing care. Public welfare services and market-oriented services are only a supplement. Many of the laws related to social security take the family and family support relationship as a precondition. However, intergenerational relations and intergenerational conflicts are unavoidable problems in Chinese society. Although family care can make it easier for the elderly to feel family and spiritual comfort, it can also internalize intergenerational conflicts. Therefore, the old-age care transition from the family to the society can alleviate the contradiction between the elderly and the young population within the family. Adopting a community-based care model can also take care of the elderly's desire to live in a familiar environment and enjoy their old age (2).

Second, the problem of aging in China is increasing, and the government alone cannot solve the problem fundamentally. It is imperative to develop a diversified model. Timebank age care services can arouse the enthusiasm of community residents and attract volunteers to participate, which is a useful supplement to the home care model (3). The reason is that this model can provide a way for some elderly people with low incomes and insufficient ability to purchase care services independently, which is conducive to saving the social cost of pensions and effectively alleviating the financial demand pressure of elderly services.

Third, after the low-age elderly withdraw from the job market according to the national legal retirement ages of 55 years old for man and 50 years old for woman, a large part of them still have the potential to continue working. However, due to insufficient physical conditions, knowledge, and skills, most of the low-age elderly can only use their spare time to serve the other elderly in the community (4). Therefore, the timebank model can use market-oriented means to mobilize their enthusiasm. Fully basing on the existing social human resources, especially the human resources of low-wage elderly to broaden the group of elderly service personnel, can solve the problem of insufficient elderly service personnel caused by the aging society under Chinese huge population base.

Fourth, the needs of the elderly in China are transforming from low-level and single to high-level and diversified. The focuses of the elderly care problem are not only the satisfaction of the basic living needs of the elderly and the guarantee of money alone but also the development of a higher level of diversification in the Chinese present situation. The timebank can play a positive role in alleviating this contradiction between supply and demand through the “timesaving” mechanism in a broad population group (5).

At present, there are many problems in the development of the timebank model in China. First, the low popularity and small scale of existing timebanks hinder the transfer and conversion of time currency (6). Second, the young generation's participation is quite low in most of the Chinese timebanks (7). Third, the lack of transfer mechanism and the difficulty of general deposit and withdrawal are common problems for Chinese timebanks (8). However, the main reasons for the lagging and insufficient development of China's timebank are the lack of relevant policy support from the government, the lack of a unified network information system, and the lack of a service value measurement standard that is suitable for China.

### Operation Mode of Chinese Timebank

The earliest timebank model in China appeared in the Jinyang Neighborhood Committee of Tilanqiao Street, Hongkou District, Shanghai in 1998, which called on the young elderly to provide services for the other elderly. The earliest “time savings card,” also appeared in the Jinyang neighborhood committee, which includes labor time savings date, content, and time. At present, the development of the timebank is no longer limited to the big cities along the eastern coast, but further expanded to Hubei, Inner Mongolia, Sichuan, Guizhou, and other inland provinces, though, almost all the time banks are small community time banks. This paper takes the Zhaoyuan community timebank in Nanjing, a rapidly developing one, as an example to illustrate the typical operation mode of China's timebank at present.

In August 2005, a community timebank was set up in the Zhaoyuan community in Nanjing. The idea behind the timebank is that the elderly population in the community is growing, and most of the younger ones are idle. By the end of 2020, the number of members has reached more than 2,000 people, most of whom are retired old people in the community, as well as college students practicing volunteer service. In the process of continuous exploration and operation, the Zhaoyuan community timebank has gradually formed its own complete operating system.

#### Relevant Supporting Institutions

Zhaoyuan timebank uses the time currency to record the accumulative service of its members and sets up corresponding institutions to ensure its normal operation, including time currency maintenance and management department, time currency evaluation and arbitration department, time currency coordination and development Department, and time currency credit and trading department.

#### Contents of the Service Provided

Zhaoyuan timebank not only provides services for the elderly, but also help the vulnerable groups in the community, such as the seriously ill, the injured, and the mentally ill. Now there are more than 30 service items including household appliances, computers, and bicycle repair, hairdressing, health consultation, housekeeping services, legal aid, elderly psychological counseling, emergency patient rescue, on-site physical examination, photography, flower care, marriage introduction, cultural and recreational activities and so on, which basically include every aspect of daily life needs for the community elderly residents.

#### Selection of Members

In addition to the elderly and other poor groups, the general people to enjoy the service of Zhaoyuan timebank must first accumulate 60 h of time currency. In addition, according to the cumulative length of time currency, the participator will get the star rating. Zhaoyuan community timebank divide the participators into five levels of member honor, and corresponding reward measures are given for each honor level.

#### Timebank Credit System

The time currency can be overdrawn, that is, if the time currency accumulation is not enough, or no accumulation when meeting the need for emergency services, the members can enjoy the service by paying a certain amount of security fund, and then provide services for others to offset the overdraft. The timebank also set up a families guarantee system, which is also used control to overdraft risk.

### Literature Review

Timebank, like traditional banks, is the service institution that supplies to clients store and withdrawal service of laboring time. The researches on timebank mainly focused on three aspects, including operation mechanism, participation willingness, and development situation of timebank. However, each aspect is in its early study period and their conclusions are inconsistent.

For the studies of the operation mechanism of timebank, scholars have, respectively, analyzed different problems like credit, labor supply, and demand distribution. The new pattern of old-age care can be explored according to the operation of the timebank under the new sharing economy (9). It can be a prominent non-profit counterpart of commercial peer-to-peer service exchange businesses, such as Airbnb, Lyft, and TaskRabbit, which are expanding rapidly (10). It is also a “currency community” which uses the hour as the unit of exchange, where every single person's input is valued equally (11). Timebank's most commonly cited benefits are building social capital and empowering members (12). It is found to be successful at engaging socially excluded and vulnerable groups of people in community activities, boosting their confidence, social networks, skills, and well-being (13). Furthermore, timebank developed a significant communication and social network that members activated to solve diverse practical problems facing the community (14). It offers a great opportunity for solving various intercultural issues, especially by enabling the skills and offer-demand based exchange without taking the cultural background much into consideration (15). For example, based on three cases study, Dash and Sandhu found that the obstacles can be addressed by hybrid models between timebank and volunteering efforts (16). Based on the data collected from timebanks in three European metropolitan areas, Laamanen et al. considered that timebank as a collaborative consumption lifestyle that challenges the traditional monetized ideology of exchange in orthodox economic theory and the hegemonic understandings of consumption (17). Besides, based on a 2013 ruling by the Finnish tax authority and the Timebank's responses to it, Matti concluded that to what degree the choice of a particular standard can be taken as a moral choice (18). For the studies on China, Zheng introduced the concept of time currency to timebank, which is mainly issued by the government, as the accounting unit, transaction currency, payment currency, and deposit currency of timebank services. According to the actual situation of the elderly, the government grants time and currency subsidies as appropriate, which greatly promotes the development of timebank (19). From the perspective of aging, Lu and Wu do not regard the elderly as a burden to society, but one of the valuable social labors who contribute to society. Timebank can let the elderly better integrate into the political, economic, technological, and cultural development of society, and provide their own strength for social development (20). Based on the above, we set up relevant questions in the questionnaire to investigate the willingness of the elderly in the community to provide services through an online timebank.

For the studies of the people's willingness to use timebank, in addition to the middle-aged and elderly as the main body of aging, there are also community volunteers such as young people who are willing to participate in the healthcare services through timebank. For example, by introducing a timebank smartphone application and present a 5-week user study with 32 young adults, Han et al. highlighted the potential of timebank for the young population with an application that facilitates access to communications and transaction-management activities and strengthens social connection and the sense of community attachment (21). Based on 87 months' worth of transaction data from a timebank, which has had a total of 950 members, the elderly were found to be as active as other members (22). Besides, how to improve the willingness to participate in timebank is also a topic of concern for scholars. A movement is gaining traction in New Zealand around timebanks, networks of support in which members exchange favors such as gardening, lifts to the supermarket, pet care, language lessons, career advice, or smartphone tutorials (23). By analyzing a combination of service exchange records from the three largest timebanks in the world, Shih et al. suggested that the idea of “equal time, equal value” that is at the foundation of timebank is a source of tension between members with instrumental vs. idealistic and altruistic motivations (24). Some scholars have also found that online timebank can increase the willingness to participate. For example, based on the survey study over 120 timebanks across the USA, Chien et.al. found that perceived ease of use in timebank platforms was positively associated with positive attitudes toward both requests and offers (25). By exploring the potential of an online timebank, Nind et al. consider that, while the concept is some way from becoming a reality, a hybrid digital-physical timebank, if accessible and flexible enough to attract usage, has the potential for supporting democratized, inclusive research in practice (26). For the studies on China, Ding took urban retired healthy elderly people as the research object, investigated the willingness of this part of the population to participate in timebank and found that the willingness of urban retired healthy elderly to participate in timebank has reached about 40%. It is believed that more and more people will recognize and accept the social age care service model especially the timebank (27). Zhang and Han explored the feasibility of constructing a “shared living model.” They believe that the concept of “Internet +” can be used as a link to join the elderly and young people. In addition to the traditional youth providing services and the elderly enjoying services, they also proposed a two-way supply-demand relationship in which the elderly impart experience and knowledge to young people (28).

For the studies of the development situation of timebank, the low popularity and small scale of development hinder the transfer and exchange of time currency. For example, based on the Data collected from interviews from 84 timebank members, and semi-structured interviews with 13 timebank staff, Ruth et al. found that Timebanks faced significant implementation challenges including managing risk and safeguarding and the associated bureaucracy, a paternalistic professional culture and the complexity of the timebank mechanism which required adequate resources (29). By investigating 892 community residents' willingness and their perception of timebank in Hangzhou, Shao et al. considered that we should strengthen the application of intelligent technology to enhancing the ability to meet the demand of mutual volunteer assistance for the elderly, and perfecting the timebank model to enhance the sustainable development ability of mutual volunteer service (30). In addition, Hooper et al. introduced a kind of social machine, which is an online timebank and a time-based way for people with learning disabilities to give and receive services (31). For the studies on China, after summarizing the innovations of China's timebank, Chen and Huang concluded that the shortcomings of the timebank's early development mainly include narrow community coverage, the value measurement problem of specific services, the operation problem of timebank and especially the low participation of the young generation (7). Qi and Gao studied the problems encountered in the development of timebank and pointed out that the government's superstructure support is the main shortcoming. At the same time, there are many other problems such as the value measurement of service items, the time savings of population changes, the lack of transfer mechanisms, and the difficulty of universal deposit and withdrawal (8).

In summary, the literature has no questionnaire investigation on the willingness of online timebank participation and its influencing factors, especially for China where the development of timebanks lags behind. Therefore, this study uses questionnaires for the young and the elderly to investigate the potential elderly care needs, participation willingness and feasibility of the online timebank healthcare model, and discover the problems in the development of timebank thus putting forward specific countermeasures and policy suggestions.

## The Survey Process and Data Analysis

### Questionnaire Design

Based on the current situation of the rapid development of the internet industry, this article conducted a questionnaire survey among people of two different ages for internet timebank participation willingness. This survey was conducted in October 2019 and divided people of different ages into youth group under 50 years old and elderly group over 50 years old. For the two groups, different questionnaires are designed and issued for different questions such as gender, age, occupation, and income to reflect the influence of different factors on people's willingness to participate in the online timebank.

In this questionnaire, a total of 16 questions were set in the young group, including 6 basic questions such as gender, age, and occupation in the beginning. After that, there are four questions about the daily arrangement of time and the basic situation of the elderly at home, mainly including the daily leisure time of the elderly at home, the number of the elderly, and what they do in the leisure time. Through these questions, the free time of the elderly and their activities in free time can be effectively understood, so as to better integrate these activities into the time bank system. Questions 11–14 analyze the implementation process and the relationship between supply and demand of time bank by understanding the young people's willingness to serve the elderly and what kind of return they hope to get. The last two questions directly focus on the concept of the online timebank, and investigate the conversing methods of the online time bank, such as the time conversion for different labor intensity standards and time and currency conversion standards.

For the elderly group, a total of 19 questions were set, including eight basic questions in the beginning. According to the characteristics of the elderly, two questions were added in the basic ones, namely whether they retired or not and their health status. Questions 9–11 mainly investigate what the elderly do in their leisure time, so as to know their needs more accurately. Questions 12–17, from the perspective of public welfare, investigate the attitudes and views of the elderly toward the time bank, and at the same time investigate the multi-level needs of the elderly, that is, the spiritual needs of the elderly in addition to the living needs, and the work needs of the elderly after retirement, so as to better develop the human resources, especially the human resources of the young elderly. Finally, questions 18-19, like the young group, analyze the acceptance of different pricing methods of online time banking and the acceptable conversion standard of time and currency in the online timebank.

### Investigation Process

The investigation was conducted in groups in 2019. The investigation of the young group was conducted by the online questionnaire program, which was promoted by the social tool WeChat. However, the online questionnaire program is hard to operate in the elderly group. Therefore, we took the offline investigation. There are six members in our investigation team. We went to the main parks, large communities, and vegetable and fruit areas of some supermarkets in Beijing separately in groups of two to conduct one-on-one interviews and questionnaire survey with the elderly randomly. Among them, what impresses us more is that two elderly people with relatively higher education (referred to here as A and B) hold opposite attitudes toward timebank, which has caught our attention.

Both Mr. A and Mr. B have a master's degree or above. After we explained our purpose, Mr. A gave a very positive attitude toward our investigation work, carefully understood our research topic, and expressed some concern about the aging population. Mr. A believes that the model of time bank is worth popularizing, and he has affirmed the possible activities that the elderly want to participate in our questionnaire survey. Here is a summary of Mr. A's thoughts on the Time Bank and some suggestions. Mr. A believes that the timebank, as an institution of public welfare, is easy to win the trust of the elderly. Second, the model of time exchange in the time bank effectively use the old labor surplus. For example, the old people's skills and experience can be used to help their neighbors solve problems, and sometimes they can also help the neighbors who go to work to do some physical labor within his capacity. These not only affirm the value of the elderly, but also improve their happiness. Mr. A said he is willing to use his knowledge and skills to help more people. Third, the model of time bank also reflects the traditional Chinese moral concept—filial piety comes first. Young people's efforts provide time storage for the elderly and provide different ways to express their filial piety to the elderly. However, Mr. A put forward that the tim bank model also has a lot of normative problem and security problem. The first one is the institutional guarantee. Old people pay more attention to if there are any support and encouragement from the government. If there is national support or national coercive protection, it will give the elderly a considerable degree of security and make them trust the time bank more. Second, as for the security issue of timebank, just like the new thing insurance that was unknown in the 1980s, how does it maintain the protection of people's labor? How is the time store in the time bank recorded and protected? This is also an area where it takes time for the time banking institutions to be further improved. Third, regarding the implementation of online timebank, it is necessary to make relevant planning and design for the elderly users who are not accustomed to using computers and mobile phones to participate in time bank more conveniently. Finally, there is the issue of the publicity of the timebank. Although there are a small number of time banks in first-tier cities such as Beijing, Shanghai, Guangzhou, and Shenzhen, they have not formed a considerable scale, and the common people are not familiar with the concept of time bank itself. Getting people to accept such a new concept in a short period of time seems to be the first issue we need to consider in developing the timebank.

Mr. B, on the other hand, holds a completely different view from Mr. A. He believes that when he is strong, he works and lives in a step-by-step manner and nine-to-five jobs. After retirement, he hopes to live at his own pace and enjoy the freedom of time. Therefore, he does not want to participate in community activities, including time banking activities, to accumulate time for his future care. Mr. B hopes that he can travel or do the things he likes, and enjoy life like idle clouds and wild cranes. This situation is not unique. Among the old people, we interviewed in different areas, there are always about 1/3 of them who want to live such a life. It is not just that they reject the time bank model, but that they prefer the freedom of old age life after being “tied down by work” for a long time. Therefore, when promoting the timebank, we should make corresponding propaganda countermeasures for these elderly people. For example, it is not only to save time for their own aged care but also a kind of public welfare activity to help others and increase social communication with community neighbors.

In the course of the investigation, in addition to answering the questions of the questionnaire, some of the elderly people we randomly interviewed also asked us about the implementation method and future development direction of time banking in detail and expressed great expectations for the online development of time bank in particular.

### Data Analysis

The elderly group received 115 valid questionnaires, and the young group received 238 valid questionnaires. The sample size is not high for the following two reasons. First, although the online questionnaire survey method is adopted in the young people's group, a few of timebanks founded by some individuals are only scattered in some big cities with a high aging population, and most of the young generation are not clear about the concept of timebank, so they cannot carry out a complete and valid questionnaire. Second, in the process of using a face-to-face interview questionnaire survey for the elderly, it was found that many people did not believe in this mechanism because they could not understand it or had no contact with it. They were unwilling to participate in and refused to accept the investigation. Therefore, it was difficult to collect samples on the whole. However, if the sample size is more than 10 times of the independent variables, generally the Logit regression result has good explanatory power. Therefore, the sample size in this paper meets the standard of the sample size of the Logit model (32).

**Figure 1:**
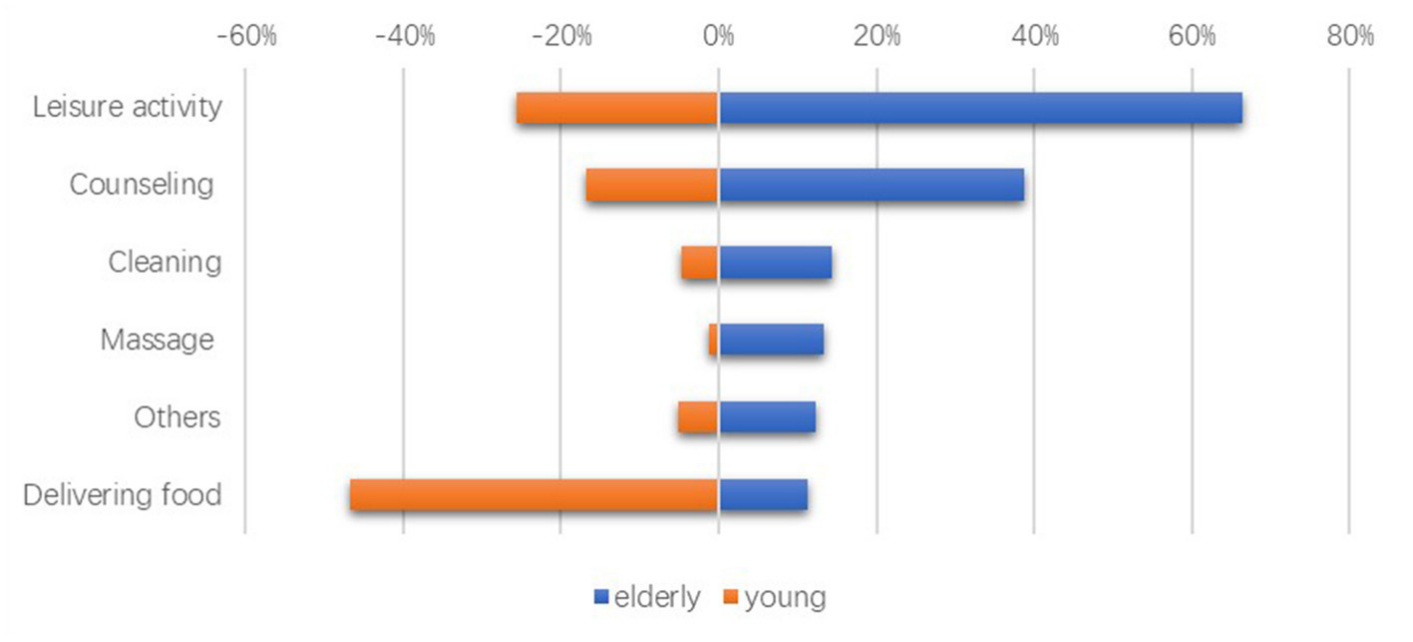
The elderly demands and the young supplies. The unit is number.

First, before studying the impact of different factors on timebank's participation willingness, it should also pay attention to whether the demand and supply of services match. Based on the survey data, we found that leisure and recreational activities and seminars and consultation sessions are more in demand by the elderly, and these are also services that young people are more willing to provide. Although more than 80% of young people are willing to participate in the delivery of food and fruit, which ranks first, it is the service that the elderly need least. This shows that online timebank may play an important role in matching the demanded services and supplied services. The above findings are indicated in Figure 1.

**Figure 2:**
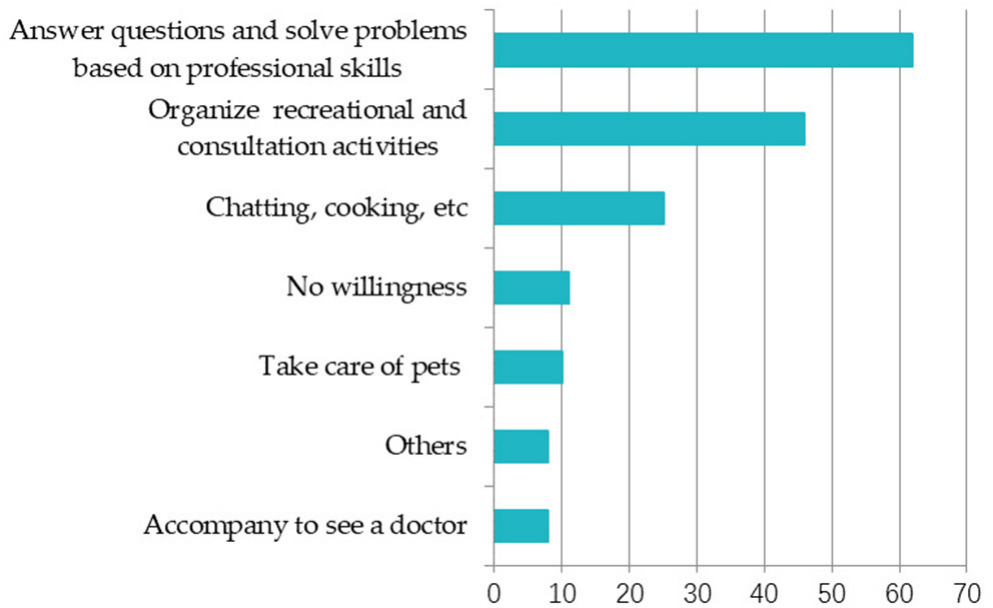
Services that the elderly are willing to provide. The respondent can choose more than one; the unit is number.

Second, as shown in Figure 2, it should also be noted that the elderly can also serve as the suppliers to provide corresponding services to other elderly people in the community, which can enable their social value to be embodied to a higher degree while also meeting the psychological needs of the elderly themselves. While providing the services to the elderly as those indicated in Figure 2, timebank can make full use of the elderly's own experience and talents to achieve the goal of “I am for everyone, everyone is for me.”

**Figure 3:**
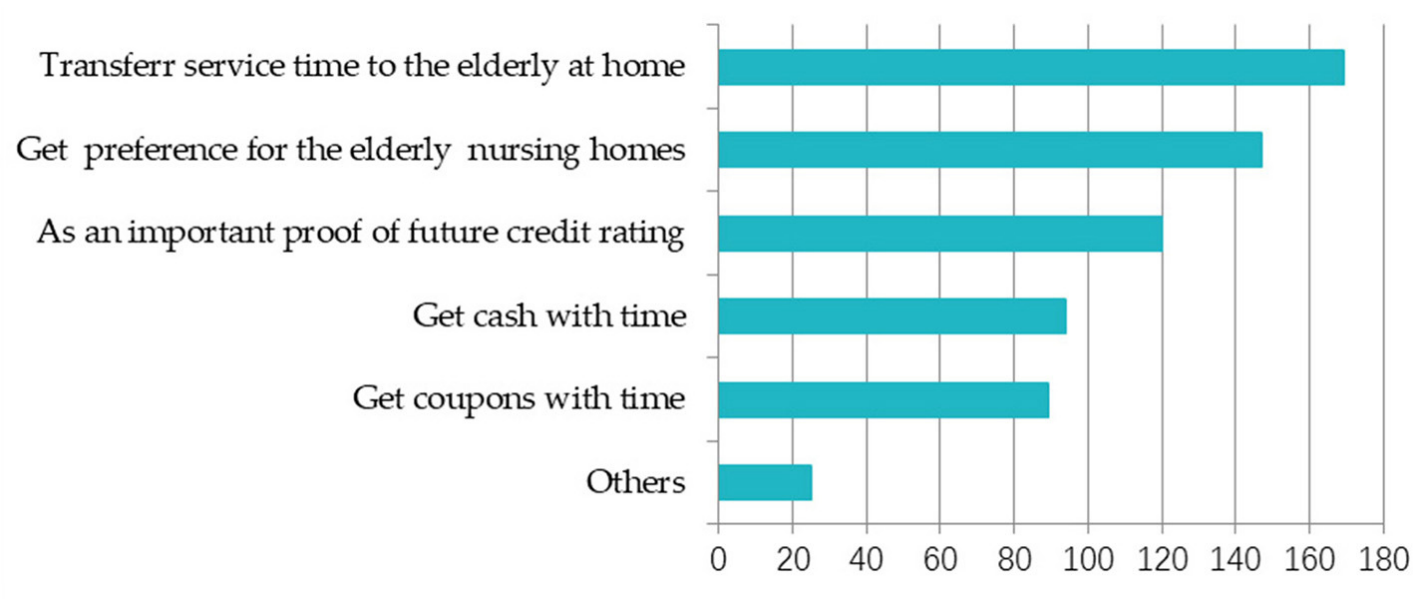
The benefits that young group hope to get. The respondent can choose more than one; the unit is number.

**Figure 4:**
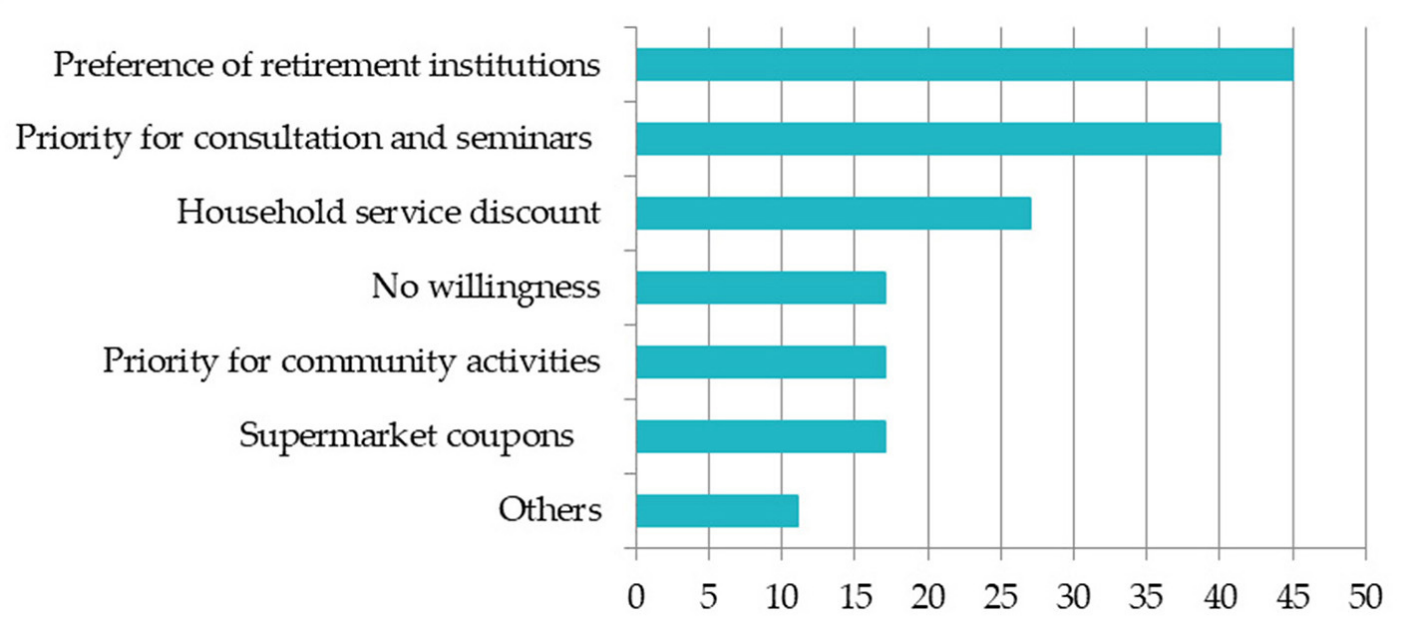
The benefits that elderly group hope to get. The respondent can choose more than one; the unit is number.

Third, in general, the incentive mechanism of timebank is relatively limited because of its non-profit nature. In Figures 3, 4, it was found that transferring time to elderly families in different ways is the most popular way of benefits, with 70% of people willing. The second is as a credit rating certificate and to obtain cash exchange or coupons. For young people, due to the large time span and long redemption time, young people are more willing to transfer their reserve time to elderly families. Therefore, government credit endorsement and transferability of service time are very conducive to the development of timebank.

## Methods

### The Logit Model

Based on the researches of previous scholars and combined with the characteristics of this research, we select the logit model in the empirical analysis. The logit model is also called the logistic regression model, which is one of the discrete choice method models and a category of multivariate analysis. It is widely used in various decision analysis because its probability expression is explicit, the solution speed of the model is fast, and the application is more convenient. When there is no change in the selection set of the model but the level of each variable, we can easily solve the selected probability of each selection branch in the new environment. According to the characteristics of the logit model, any increase or decrease of choices will not affect the ratio of the probability of being selected among other choices, so it is possible to directly remove the choices that need to be removed from the model, or add the newly added choice branches to model for prediction. When the dependent variable is a binary variable, such as “whether you will use online timebank as the care model,” logit distribution can be used as the distribution function of the classified variable. The logit function and logit regression model are as follows:

(1) $$p\left(y_{i}=1|x_{i}\right)=p\left[\varepsilon_{i}\leq \alpha +x_{i}^{\prime }\beta \right]=\frac{1}{1+\text{exp}(-\varepsilon_{i})}$$

(2) $$\begin{array}{l} \text{ln}\left(\frac{p_{i}}{1-p_{i}}\right)\;=\;\alpha +x_{i}^{\prime }\beta \end{array}$$

In the above formula, α is the intercept term, β is the regression coefficient, and *x* represents the explanatory variable for the selection of online timebank as the care model.

The logit regression model is non-linear, so the maximum likelihood estimation method is generally used to calculate the model parameters. Before using the maximum likelihood estimation method, it is necessary to establish the likelihood function obtained by expressing the probability of selecting the number of times of foreign investment as the unknown model parameter. The maximum likelihood estimation of the model parameters is the parameter estimation value that can maximize the value of this function. The derivation process is as follows:

We assume that the probability of choosing online timebank as the care model is *p*(*y*_*i*_=1|*x*_*i*_)=*p*_*i*_ , then the probability of not choosing online timebank as the care model is *p*(*y*_*i*_=0|*xi*)=1-*pi*, so

(3) $$\begin{array}{l} p(y_{i})=p_{i}{\;}^{y_{i}}{\left(1-p_{i}\right)}^{1-y_{i}} \end{array}$$

Because each person makes an independent decision, through natural logarithm processing, we finally get its likelihood function:

(4) $$\begin{array}{l} \ln\left[L\left(\theta \right)\right]=\text{ln}[\prod_{i=1}^{n}p_{i}{\;}^{y_{i}}{\left(1-p_{i}\right)}^{1-y_{i}}\;]\\ \;=\sum_{i=1}^{n}\left\{y_{i}\left(\alpha +x_{i}^{\prime }\beta \right)-\text{ln}[1+\text{exp}(\alpha +x_{i}^{\prime }\beta )]\right\} \end{array}$$

After taking the logarithm of the likelihood function, the intercept term and regression coefficients of the explanatory variables are, respectively, calculated for partial derivatives, we set it to 0, and overall parameters that make the likelihood function reach the maximum can be obtained by iterative calculation.

### Variable Selection

#### Dependent Variable and Interested Variables

On the one hand, the dependent variable (***OTB***) is in the form of a dummy variable, representing whether the respondent will use online timebank as the care model. If the respondent responded yes, the value of the variable is 1; if not, the value is 0.

On the other hand, there are three key variables, respectively, in both the model of the young group and the model of the elderly group. The core variables in the elderly group are health status, voluntary experience, and daily free time. The core variables in the youth group are elderly families, voluntary experience, and daily free time.

The health status (***Hea***) of the elderly group is a dummy variable, with a value of 1 for good health status and a value of 0 for bad health status. The health status of the elderly affects whether they are able to save time as a service provider in the timebank for the aged care need in the future (16). We predicted that elderly people in good health would be more likely to participate in the timebank, and those in poor health would be less likely to participate.

Voluntary experience (***Vol***) means whether the respondent has participated in voluntary activities, which is also a dummy variable, with a value of 1 for having voluntary experience and a value of 0 for having no voluntary experience. Voluntary experience means that the respondent has volunteered to help others and has a charity heart. The people with voluntary experience is more motivated to participate in a timebank that may exist in a pro bono nature.

The elderly families of the young group (***Eld***) mean the number of elderly families over 55. The more elderly families young people have, the heavier the burden they have to support or take care of the elderly families, and the more willing they are to participate in the timebank and transfer their reserve time to the elderly families (28).

Daily free time (***Time***) means how much free time the respondent has each day. The more the free time they have every day, the more they are inclined to participate in timebank for time saving. We predicted that it has a positive effect on the online timebank participation willingness in both the elderly group and the young group.

In addition, the control variables representing the other influencing factors of participation in online timebank nursing for community elderly are divided into two aspects: individual and employment. Variables representing individual characteristics are gender, political status, age, and education years; variables representing employment characteristics are occupational income and job type. The following two sections will describe the control variables in detail.

#### Control Variables for Individual Characteristics

The age (***Age***) may have a significant effect on the online timebank participation willingness in both the elderly group and the young group, but its effect is unclear. The younger they are, the more likely they are to accept and use the online time-banking care model, but they lack their own age care needs and their elderly families are not very old. The older they are, the higher their demand for future care needs will be, but they are not easy to accept and use the new age care model of online timebank. Therefore, we do not prejudge the effect of age on participation willingness.

Gender (***Gen***) is a dummy variable, with a value of 1 for the male and a value of 0 for the female to reflect the gender difference in participation willingness of online timebank. There may be gender differences in the acceptance degree of the online timebank model, but its effect is unclear. Therefore, we do not prejudge the effect of gender on participation willingness.

Political status (***Pol***), with a value of 1 representing party membership, and a value of 0 representing non-party membership. Party membership has a positive effect on the participation willingness of the timebank due to it has somewhat public welfare nature. We predicted that it has a positive effect on the online timebank participation willingness in both the elderly group and the young group.

The education years (***Edu***) represent the educational level of the respondent. Education may have a positive influence on the online timebank participation willingness because the higher the education level is, the higher the acceptance of new things and the internet. According to the definition of the average years of education by the China Bureau of Statistics, we divide years of education into 6 levels: a value of 0 represents those who have not received any education; a value of 6 represents those who have graduated from primary school; a value of 9 means people who have obtained a junior high school education; a value of 12 represents four types of people, that is, people with an education level of ordinary high school, vocational high school, technical secondary school, or technical school; similarly, the value of 16 represents four types of people, that is, those who have obtained adult advanced education and formal higher education include those with a college degree or a bachelor's degree; the value 19 represents the population with a graduate degree or above. We predict that education years have a positive effect on online timebank participation willingness in both the elderly group and young group.

#### Control Variables for Employment Characteristics

As the income level, occupational income (***Inc***) represents the income from the current employment. Generally, the higher income of the occupational income is, the lower needs for online timebank participation because they can afford the relatively expensive nursing fees like Nursing Home. Therefore, we predicted that it has a positive effect on the online timebank participation willingness in both the elderly group and the young group.

Job ownership (***Job***) means the ownership of the employer including government and public institutions, state-owned enterprise, and private and foreign companies. Influenced by different corporate cultures, Chinese people working in enterprises of different ownership may have different degrees of acceptability to new things. On the whole, the government and state-owned employees are more conservative, while foreign and private employees may be more open and inclusive regarding online timebank.

To sum up, Tables 1, 2 show all the variable definitions in the elderly group and the young group as follows.

**Table 1:** Variable definition: the elderly group.

**Variable**	**Definition**	**Classification**	**Percent**
***OTB***	Whether you will use online timebank, 1= yes, 0= no	Yes: 60 No: 55	53.91% 46.09%
***Gender***	1= male 0= female	Male: 60 Female: 55	52.17% 47.83%
***Age***	Unit is year	Mean=56.8	
***Inc***	Monthly income, Unit is RMB	Below 5,000^*^: 10 5,000–10,000: 49 10,000–15,000: 39 Above 15,000: 17	8.70% 42.61% 33.91% 14.78%
***Pol***	Whether the respondent is the Party member	Yes: 51 No: 64	44.35% 55.65%
***Job***	The ownship of the employer	Government: 65 State-owned: 19 Private or foreign^*^: 31	56.52% 16.53% 26.95%
***Hea***	Health status of respondent, good = 1, bad = 0	Good: 101 Bad: 14	87.82% 12.18%
***Vol***	Whether you have participated in volunteer activities before	Yes: 41 No: 74	35.65% 64.35%
***Time***	Free time every day	<2 h^*^: 25 3–5 h: 47 More than 6 h: 43	21.74% 40.87% 37.39%
***Edu***	Years of education	6 years: 9 9 years: 13 12 years: 24 16 years: 65 19 years: 4	7.83% 11.31% 20.86% 56.51% 3.49%

The * means that it is the benchmark. The percentage retains 2 decimal places. The length of education is converted by education level, specifically 6 years of elementary school, 9 years of junior high school, 12 years of high school, 16 years of university, 19 years of postgraduate, and above.

**Table 2:** Variable definition: The youth group.

**Variable**	**Definition**	**Classification**	**Percent**
***OTB***	Whether you will use online timebank, 1 = yes, 0 = no	Yes: 145 No: 93	60.62% 39.38%
***Gender***	1 = male 0 = female	Male: 131 Female: 107	52.17% 47.83%
***Age***	Unit is year	Mean = 33.6	
***Inc***	Monthly income, Unit is RMB	Below 5,000^*^: 71 5,000–10,000: 72 10,000–15,000: 59 Above 15,000: 36	29.83% 30.25% 24.79% 15.13%
***Pol***	Whether the respondent is the Party member	Yes: 83 No: 155	34.87% 65.13%
***Job***	The ownship of the employer or the respondent is student	Government: 49 State-owned: 29 Private or foreign: 82 Student^*^: 78	20.59% 12.18% 34.45% 32.78%
***Eld***	Elder families number over 55	Mean = 2.8	
***Vol***	Whether you have participated in volunteer activities before	Yes = 141 No = 97	59.24% 40.76%
***Time***	Free time every day	<2 h^*^: 87 3–5 h: 100 More than 6 h: 51	36.55% 42.02% 21.43%
***Edu***	Years of education	6 years: 0 9 years: 15 12 years: 20 16 years: 122 19 years: 81	0% 6.3% 8.4% 51.26% 34.04%

The * means that it is the benchmark. This survey also involves the health status of the youth group, and it is found that nearly 98% of them are in good health, so it is not used as an independent variable for empirical analysis.

### Model Specification

Based on the above analyses and the variables selected, the following models are specified:

(5) $$\begin{array}{l} OTB_{i}=\beta_{0}+\beta_{1}Hea_{i}+\beta_{2}Vol_{i}+\beta_{3}Time_{i}+\beta_{4}Age_{i}+\beta_{5}Gen_{i}\\ \begin{array}{l} \;+B_{6}Pol_{i}+B_{7}Edu_{i}+B_{8}Inc_{i}+B_{9}Job_{i}+u_{i} \end{array} \end{array}$$

(6) $$\begin{array}{l} OTB_{j}=\beta_{0}+\beta_{1}Eld_{j}+\beta_{2}Vol_{j}+\beta_{3}Time_{j}+\beta_{4}Age_{j}+\beta_{5}Gen_{j}\\ \begin{array}{l} \;+B_{6}Pol_{j}+B_{7}Edu_{j}+B_{8}Inc_{j}+B_{9}Job_{j}+u_{j} \end{array} \end{array}$$

where *i* (i = 1, 2, …, 115) denotes the elderly respondent and *j* (j = 1, 2, …, 238) denotes the young respondent; *OTB* indicates online timebank participation willingness; *Hea* in model 1 indicates the health status of the elderly respondent and *Eld* in model 2 means the number of elderly families of the young respondent; *Vol* indicates the voluntary experience; *Time* indicates the free time every day; *Age* indicates the age of the respondent; *Gen* means the respondent's gender; *Pol* represents whether the respondent is the Party member; *Edu* is the educational years of the respondent; *Inc* is the annual income from the employer; *Job* indicates the employer ownership, and *u* is a random disturbance term. Equations (5) and (6) allow us to test the impacts of various factors on the online timebank participation willingness in both the elderly group and the young group.

## Exploring the Influencing Factors of Participation Willingness

### Regression Results Analysis

As shown in Table 3, the results of model 1 is for the elderly group and the results of model 2 is for the young group. Given other variables remain unchanged, the logit regression results can indicate the following information based on the independent variables in which we are interested.

**Table 3:** Logit regression results: by groups.

**Independent variable**	**Dependent variable:** ***OTB***	
	**Model 1: The elderly Group**	**Model 2: The young group**
***Eld***		0.041^***^ (0.0013)
***Hea***	0.368^**^ (0.0443)	
***Vol*** ***Time 1 (3–5 h)*** ***Time 2 (Above 6 h)*** ***Gen*** ***Age***	0.012^***^ (0.0000) −0.021^***^ (0.0000) 0.064^**^ (0.0250) 0.031^***^ (0.0002) −0.003^***^ (0.0000)	0.053^***^ (0.0000) 0.022^***^ (0.0020) 0.074^***^ (0.0050) −0.038 (0.5002) −0.031 (0.5030)
***Pol***	0.021^**^ (0.0124)	0.010^***^ (0.0000)
***Edu***	0.081^***^ (0.0004)	0.106^***^ (0.0000)
***Job 1 (government)***	−0.002 (0.1135)	−0.090^*^(0.0925)
***Job 2 (State-owned)***	−0.003 (0.1420)	−0.072^*^(0.0611)
***Job 3 (Private and foreign)***		−0.043 (0.1522)
***Inc 1 (5000-10,000)***	0.013^**^ (0.0420)	0.014 (0.1560)
***Inc 2 (10,000-15,000)***	0.004 (0.1320)	0.064 (0.3210)
***Inc 3 (Above 15,000)***	−0.023^**^ (0.0412)	0.083 (0.4311)
***Constant***	7.013^***^ (0.0000)	6.013^***^ (0.0000)
Pseudo *R*^2^ *N*	0.4552 115	0.6062 238

The P-value in brackets, *, **, ^***^ indicate statistically significant at the levels of 10, 5, and 1%, respectively.

First, the health status of elderly people is significantly and positively correlated with their willingness to participate in online timebank. When other variables remain unchanged, the probability that the elderly people in good health are willing to participate in online timebank will increase by 36%, and the regression coefficient is the highest among all variables. As the elderly people in good health account for 87.8% of the sample, this conclusion once again confirms that the elderly can also serve as the suppliers of the online timebank elderly healthcare service. Through the online platform, the elderly can make full use of their spare time and experience skills to provide corresponding services to other elderly people in the community.

Second, the number of direct elder families of young people has a significant positive impact on the willingness to participate in online timebank. When other variables remain unchanged, for every increase in the number of direct elder families, the probability that young people are willing to participate in online timebank will increase by 4.1%. This result shows that under the trend of increasing aging, timebank can be used as an effective means under the home care model, which is accepted by young people and meets the urgent needs of the elderly.

Third, the experience of participating in voluntary activities has a significant positive effect on the willingness to participate in the online timebank of the young group. The experience of participating in volunteer activities has a far greater impact in the young group than that in the elderly group because the regression coefficient in the young group is close to 5 times as much as that in the elderly group. When other variables remain unchanged, the young group who has participated in voluntary activities will increase the probability of participating in online timebank services by 5.3%, while the elderly group will only increase by 1.2%.

Fourth, for the elderly group, the free time every day has different effects on the willingness to participate in online timebank. Three to five hours of free time has a significantly positive effect on the willingness to participate in online timebank in the elderly group. Free time of 6 h or more has a significantly negative effect on the willingness to participate in online timebank in the elderly group. The possible reason is that according to the situation in China about 70 percent of children care are assisted by elderly family members, there is not much free time for the elderly^2^. The elderly people are more inclined to take care of their grandchildren or develop their own hobbies and activities and maybe they have no spare time to participate in online timebank. When the free time reaches more than 6 h per day, elderly people can guarantee the time to participate in other extra activities such as online timebank healthcare activities. For the young group, the variable *Time 1* (3–5 h free time) with the coefficient of 0.021 and the variable *Time 2* (above 6 h free time) with the coefficient of 0.074 both have significantly positive effects on the participation willingness in online timebank.

For the control variables, the results are quite different between the elderly group and the young group. In the elderly group, the male is significantly more willing to participate in online timebank than the female, but in the young group, it is statistically insignificant. The reason may be that Chinese elderly females have relatively conservative thought and the timebank service is relatively not safe for them. Age has a significantly negative impact on the participation willingness of the elderly in online timebank, but in the young group, it is also statistically insignificant. The reason may be also that the older the respondent gets, the more conservative he or she becomes. In the elderly group, the variable of monthly income between 5,000 and 10,000 RMB has a significantly positive impact on the participation willingness and the variable of monthly income above 15,000 RMB has a significantly negative impact on the participation willingness in online timebank, which may be due to that the elderly people with higher incomes can afford institutional care fees without having to participate in a timebank. In the young group, the people who work for the government, state-owned enterprises, and private and foreign enterprises are less willing to participate in the online timebank than the students, which may be due to their higher income and less free time than that of students. Besides, the party members and the educational years both have significant positive impacts on the participation willingness in online timebank, which conform to our predictions.

### Robustness Check

We conducted the following robustness check for the regression results.

First, through non-linear OLS regression, as shown in Table 4, we compare the results between the two models. It shows that the significance of the core independent variables and most of the control variables are consistent with the previous Logit model results. The *R*^2^-values of the OLS model are 0.38 and 0.41 in model 3 for the elderly group and model 4 for the young group, respectively, while the *R*^2^ of the logit model is 0.45 and 0.60, with Wald value 71.6 and the corresponding *P*-value 0.00, so the overall equation of the logit regression model is more jointly significant.

**Table 4:** Non-linear OLS estimation results.

**Independent variable**	**Dependent variable:** ***OTB***	
	**Model 3: The elderly group**	**Model 4: The young group**
***Hea*** ***Eld*** ***Vol*** ***Time 1*** ***Time 2*** ***Gen***	0.338^**^ (0.0324) 0.012^***^ (0.0001) −0.006^***^ (0.0000) 0.002^***^ (0.0000) 0.013^***^ (0.0000)	0.036^***^ (0.0010) 0.042^***^ (0.0000) 0.024^***^ (0.0000) 0.006^***^ (0.0002) 0.062 (0.3420)
***Age***	−0.019^***^ (0.0009)	0.022 (0.2421)
***Pol***	0.007^***^ (0.0004)	0.003^***^ (0.0024)
***Edu***	0.018^***^ (0.0002)	0.012^***^ (0.0042)
***Job 1***	−0.006 (0.2392)	−0.072 (0.1451)
***Job 2***	−0.007^*^ (0.0946)	−0.033^*^ (0.0721)
***Job 3***		−0.024 (0.1432)
***Inc 1***	0.019^*^ (0.0972)	0.033 (0.2420)
***Inc 2***	0.007 (0.5442)	0.066 (0.2214)
***Inc 3***	−0.018^**^ (0.019)	−0.043 (0.143)
***Constant***	10.165^***^ (0.0000)	12.413^***^ (0.0000)
Pseudo *R*^2^ *N*	0.3790 115	0.4140 238

The P-value in brackets, *, **, ^***^ indicate statistically significant at the levels of 10, 5, and 1%, respectively.

Second, we conduct the multicollinearity test. We convert the model to a linear form and calculate the variance inflation factor (VIF). As shown in Table 5, all the VIF values of the two models are <2.18 and 1.96, respectively. It is generally believed that multiple collinearities exist when the VIF value is >10, or it does not exist. Therefore, it can be assumed that there are no multiple collinearities between the variables.

**Table 5:** Heteroscedasticity VIF test.

**Independent variable**	**Model 1**	**Model 2**
***Hea***	2.18	
***Eld***		1.84
***Vol***	2.09	1.96
***Time 1***	2.15	1.93
***Time 2***	2.03	1.92
***Gen***	2.05	1.90
***Age***	2.07	1.85
***Pol***	2.28	1.82
***Edu***	2.03	1.83
***Job 1***	1.95	1.96
***Job 2***	2.05	1.93
***Job 3***		1.94
***Inc 1***	1.79	1.86
***Inc 2***	1.56	1.84
***Inc 3***	1.39	1.93

Third, we conduct a cluster analysis. The number of years of education is used as the cluster unit. The logit model is regressed by clustering standard errors, and the results show that there is almost no difference with the general robust standard errors. It is considered that there is no correlation within the group. Due to space limitation, we do not display here.

After conducting the above robustness tests, this paper further calculated that the logit model is correctly predicted at 87.02%. In summary, it is determined that the models have robust regression results.

## Discussions

First of all, although education level has a significant impact on the participation willingness of the elderly, in the survey process we also found that it actually presents a polarized effect, which means for the elderly with a degree below the elementary school, or a graduate degree or above, the participation willingness is lower than those with an intermediate education level. Regarding this phenomenon, we interviewed some elderly people, and the reason is that elderly people with lower education level do not accept the form of online timebank because they do not understand and cannot adapt to this new age care model and even internet lifestyle. Besides, elderly people with higher education believes that in their free time, they are more willing to do what they like and do not want to be constrained by the form of online timebank.

Secondly, as for the elderly families, except for more than 5 elderly family members, the regression results show that the more elderly families, the higher the participation willingness degree. However, based on the interview, we found that there are a few families that support more than five elderly people. Compared with the online timebank age care model, these families need more comprehensive services like nursing institutions. Because of the large number of elderly people who need to be supported, it is difficult to meet the needs of community care or home care.

Third, it is found that online timebank is more likely to be favored by the elderly who like indoor activities, self-entertainment, and family entertainment. In the field interviews, some elderly people said that it would be more convenient if such activities could be made with others through online timebank. However, for the elderly who like to spend time with friends and enjoy outdoor activities, they may not have more time or willingness to participate in online timebank services. Therefore, when we operate the timebank, we can take into account the demands of these elderly people and appropriately increase outdoor activities and other activities which can be cooperated with each other.

Fourth, the analysis of the supply-demand matching found that some aged care services have high matching degrees between the elderly who need healthcare and the young who supply related services, such as organizing suitable entertainment activities and seminars and consultation meetings. However, there are some age care services such as sending fruits and other activities that are simple for young people, which are not necessary for elderly people. Through the interview, we learned that if the elderly are in good health, shopping is not only the necessary activity for daily life but also a way of socializing and exercising. Therefore, the online timebank operation mechanism should be further refined for setting up the appropriate age care item to better match the supply with the demand.

In addition, it should be admitted that there are still many deficiencies and limitations in this study.

For the limitations of the method, first, we don't take into account endogenous problems with the interested variables, such as free time, which may be associated with some unobtainable missing variables. This will be solved later by finding the appropriate instrumental variables for each of them. Second, the sampling process only involves Beijing, ignoring some other regions like the central and western regions where the economic development level is not high and the aging problem is not serious. Third, the number of participants including 115 valid elderly questionnaires and 238 valid young questionnaires in the survey sample obtained is relatively low.

For the limitations of the questionnaire, first, the settings of some questions in the survey are vague, such as the way to convert time currency to physical commodity or real money, and the way to transfer time to the elderly families, which need special research and investigation. This is also the reason why this paper only focuses on respondents' willingness to participate in online timebank. Second, the interview process for the elderly was not prepared and targeted, just recording the interviewees' views and opinions on online timebank by chatting. Third, some issues related to timebank operation were not involved in the questionnaire design stage, such as online platform privacy and security, rights and obligations of service providers and recipients and legal status, which require further data investigation and research.

## Conclusions and Policy Implications

This study uses the logit model through questionnaire data of Beijing in 2019 to investigate the participation willingness of online timebank elderly care, especially to discover different influencing factors on the participation willingness between the youth group and the elderly group. We find that: First, the health status of elderly people and the number of elder families of young people have significant positive impacts on their willingness to participate in online timebank. Second, the experience of participating in voluntary activities has a significant positive effect and it has a far greater impact in the young group than that in the elderly group. Third, the more free time the higher participation willingness in the young group, but it is the opposite in the elderly group. Fourth, for other control variables, the years of education and party member have significant promoting effects in both groups. However, compared with private or foreign companies, working for the government and public institutions and state-owned enterprises will decrease the participation probability in the young group. Besides, the results of age, gender, and income are quite different between the two groups.

Such heterogeneous results of the influencing factors can help develop online timebank nursing, especially for dealing with the increasingly serious population aging problem in China.

First, with the single-children generation gradually enters middle age, their parents are getting old. In China, it is increasingly common for a couple to support at least four of their parents. Based on the role of elderly family members in promoting participation willingness, online timebank should be promoted heavily among young people, rather than just attract the young elderly in the community to provide care services to other elderly people like the existing timebanks.

Second, generally, the retirement ages in China when a man is 55 years old and a woman is 50 years old are still relatively low. With the improvement of China's medical care and residents' health, young elderly retirees have a lot of spare power and time to serve other elderly people through timebank. Therefore, they can still serve as an important participant group in the timebank.

Third, vigorously developing social volunteer service activities, especially elderly care volunteer service activities among young people, is conducive to the participation willingness in online timebank for young people.

Fourth, in view of the positive effects of education years, student status and free time every day on the participation willingness, for young people, the promotion can be conducted among students especially the college students first. On the one hand, the college students have winter and summer vacations so there is more free time; on the other hand, the college students with high education level are more likely to accept the new concept of timebank and the age care model.

Finally, Party members in China are the main force of public welfare activities and the results show that Party members are more willing to participate in online timebank because it is somewhat of public welfare nature. Therefore, timebank can be widely publicized in China's grass-roots Party branches and some other Party organizations.

## Data Availability Statement

The raw data supporting the conclusions of this article will be made available by the authors, without undue reservation.

## Author Contributions

This research was conceptualized by CH and YW. YW and YD formulated the methodology. YW and CH: software, validation, and writing—original draft preparation. YW, YD, and LW: formal analysis. YD and LW: data curation. CH, YD, and LW: writing—review and editing and supervision. YW: project administration and funding acquisition. All authors: contributed to the article and approved the submitted version.

## Conflict of Interest

The authors declare that the research was conducted in the absence of any commercial or financial relationships that could be construed as a potential conflict of interest.

## Footnotes

^1^https://www.mps.gov.cn.

^2^China Research Center on Aging: http://www.crca.cn.
